# Salivary Exosomes in Health and Disease: Future Prospects in the Eye

**DOI:** 10.3390/ijms24076363

**Published:** 2023-03-28

**Authors:** Angela Liu, Brenna Hefley, Paulina Escandon, Sarah E. Nicholas, Dimitrios Karamichos

**Affiliations:** 1 Texas College of Osteopathic Medicine, University of North Texas Health Science Center, 3500 Camp Bowie Blvd., Fort Worth, TX 76107, USA; 2 North Texas Eye Research Institute, University of North Texas Health Science Center, 3430 Camp Bowie Blvd., Fort Worth, TX 76107, USA; 3 Department of Pharmaceutical Sciences, University of North Texas Health Science Center, 3500 Camp Bowie Blvd., Fort Worth, TX 76107, USA; 4 Department of Pharmacology and Neuroscience, University of North Texas Health Science Center, 3500 Camp Bowie Blvd., Fort Worth, TX 76107, USA

**Keywords:** salivary exosomes, exosomes, ocular disease, biomarker, wound healing, angiogenesis, inflammatory cytokines

## Abstract

Exosomes are a group of vesicles that package and transport DNA, RNA, proteins, and lipids to recipient cells. They can be derived from blood, saliva, urine, and/or other biological tissues. Their impact on several diseases, such as neurodegenerative, autoimmune, and ocular diseases, have been reported, but not fully unraveled. The exosomes that are derived from saliva are less studied, but offer significant advantages over exosomes from other sources, due to their accessibility and ease of collection. Thus, their role in the pathophysiology of diseases is largely unknown. In the context of ocular diseases, salivary exosomes have been under-utilized, thus creating an enormous gap in the literature. The current review discusses the state of exosomes research on systemic and ocular diseases and highlights the role and potential of salivary exosomes as future ocular therapeutic vehicles.

## 1. Introduction

**Figure 1:**
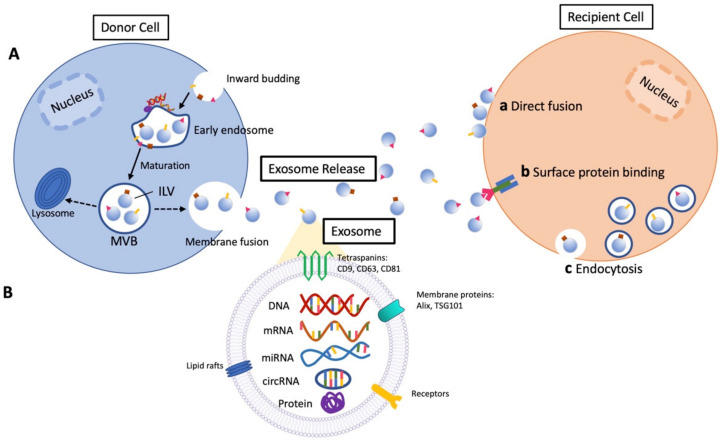
(**A**) The formation of exosomes and effects on cell–cell communication. The early endosome is formed by inward budding of the endosomal membrane and then matures to form a multivesicular body (MVB) containing intraluminal vesicles (ILVs) or exosomes. The exosome is secreted upon fusion with the cell membrane. Exosomes release their contents by (a) direct fusion, (b) surface protein binding, or (c) endocytosis. (**B**) Composition of the exosome. The exosome is a vesicle that can carry DNA, mRNA, miRNA, proteins, and lipids, etc., to modulate function of the recipient target cell.

Extracellular vesicles are membranous vesicles that are released from cells originating from the plasma membrane or endosomal system [1]. Currently, extracellular vesicles are broadly divided into three types: microvesicles, apoptotic vesicles, and exosomes [2,3,4]. Universal definitions for these three categories of extracellular vesicles do not currently exist, since the techniques to isolate and characterize extracellular vesicles properly are widely diverse and being optimized [5,6,7]. Additionally, there are size overlaps between the types of extracellular vesicles. Microvesicles, which are released from cells via the shedding of the plasma membrane, range from 100 to 1000 nm in diameter [8]. Exosomes range in size from 50 to 150 nm in diameter, making up the smallest type of extracellular vesicles [8], while apoptotic vesicles range in size from 1000 to 5000 nm in diameter [8,9]. Therefore, exosomes will henceforth be differentiated in this article by their relatively small size and further specified by their cell of origin. Exosomes are derived from endosomal membrane components and secreted after fusion with the plasma membrane [8,10], as shown in Figure 1A. After being released to the extracellular space and circulation, exosomes transfer their cargo into recipient target cells through endocytosis [11]. It is worth noting that other than the exosomes in blood circulation, most exosomes are not considered to be long-range effectors, as they are taken up by cells that are undirected in the immediate area of exosome release [11]. Through this interaction, exosomes can modify gene expression, cellular signaling, and the overall function of target cells [11]. Like their wide range of effects on recipient cells, the composition of exosomes is also diverse, containing markers including CD63, CD9, CD81, and TSG101, and essential biological molecules, such as DNA, RNA, proteins, and lipids. These components are integral to the roles of exosomes in intracellular communication and transport, as shown in Figure 1B [11,12,13,14]. Exosomes also carry IgA and plgR, which are anti-inflammatory biomarkers [15], highlighting their role in mediating the immune responses to inflammation, cell migration, and wound healing [16]. Exosomes are found in various body fluids, such as plasma [17,18,19], saliva [20,21,22], urine [23,24,25,26], and amniotic fluid [27,28,29]. Due to their versatility of function, they have gained traction over the last 20 years to serve as diagnostic biomarkers and potential treatments for numerous diseases.

Saliva offers several advantages over blood for clinical diagnosis [30,31,32]. Specifically, saliva collection is easy, non-invasive, cost-effective [31,33], does not coagulate, and is easier to handle [30,31,32]. Salivary exosomes (SEs) are being investigated as an alternative to whole saliva due to the contaminating elements and higher amylase enzyme levels that are found in whole saliva [15,34]. Additionally, the proteins and RNAs from whole saliva are susceptible to degradation when removed from their natural environment, so the protease and RNase enzymes in saliva must be stabilized with protease inhibitors and RNase inhibitors, in order to protect against the deterioration of salivary proteins and RNA [35,36,37]. Conversely, SEs, along with exosomes from other bodily fluids, have a lipid bilayer that protects their cargo, providing more accurate information for clinical diagnosis and treatment [30].

Compared to the exosomes from other bodily fluids, SEs are thought to offer a greater versatility in diagnosing and treating diseases [34,38,39]. For instance, the applications of urinary exosomes are limited to kidney and prostate pathologies, such as hyperaldosteronism [40] and prostate cancer [38,41]. On the other hand, SEs have applications in systemic diseases, in addition to organ-specific pathologies, since saliva has been reported to carry the DNA, RNA, and metabolites that are found in both the blood and saliva [30,34,39]. The advantages/disadvantages of SEs over other exosomes are summarized in Table 1.

A few drawbacks also exist with SEs [42,43]. One important factor is the inter- and intra-subject variability of the salivary flow rate and composition, depending on the method of sampling used and the subjects’ food intake, aging, and preexisting conditions [33]. Thus, the best protocol for isolating these SEs is still debatable [42,44]. A common limitation of both SEs and non-SEs is the lack of techniques to precisely quantify their molecular content [42,43,44]. Thus, understanding the components and function of exosomes in relation to pathologies is crucial to developing more disease-targeted therapies.

Due to their versatility, SEs have been studied as biomarkers for diagnosing systemic autoimmune diseases, such as oral lichen planus and inflammatory bowel disease [45,46,47]. Recently, SEs were found to carry proteins that may propagate neoplastic and neurodegenerative diseases [22,48,49,50]. However, little is known about the therapeutic effects of the SEs in ocular tissue. Here, we discuss and provide an overview of the involvement of non-SEs and SEs in systemic and ocular diseases. Each subsequent section will be organized as follows: starting with describing the pathophysiology of the disease, followed by a discussion of the non-SEs in the disease, and finally ending with a review of the SEs in the disease.

## 2. Exosomes in Non-Ocular Diseases

### 2.1. Systemic Autoimmune Diseases

The roles of exosomes in autoimmune diseases have been extensively studied. In this section, we will discuss the pathophysiology, ocular manifestations, and effects of exosomes in oral lichen planus, periodontitis, Sjogren’s syndrome, and inflammatory bowel disease [45,51,52,53], which are summarized in Table 2.

#### 2.1.1. Oral Lichen Planus

Lichen planus is a chronic inflammatory disease that is caused by T-cell proliferation and the apoptosis of the keratinocytes within the skin and mucosal membranes [54]. Oral lichen planus (OLP) occurs when inflammatory cells attack the keratinocytes within the oral mucosal lining, resulting in erythematous ulcers [55,56]. Although more rare than OLP, lichen planus may also involve ocular structures such as the eyelids, cornea, and conjunctiva, manifesting as blepharitis [57], keratitis [58], corneal perforation [59], and cicatrizing conjunctivitis [54,60,61]. To date, no exosomal studies on conjunctival lichen planus have been reported, but there are numerous reports on exosomes and oral lichen planus [62,63,64]. Plasma-derived exosomes from OLP patients were found to enhance T-cell proliferation and migration, and to upregulate pro-inflammatory cytokines such as interferon-gamma (IFN-γ), accelerating OLP progression [65]. In these studies, the plasma exosome microRNA, (miR)-301b-3p, was downregulated, while miR-130b-3p and miR-34a-5p were upregulated [62]. Interestingly, miR-34a-5p was found to be positively correlated with the severity of the clinical symptoms of OLP [62]. Yang and co-authors observed that T-cell-derived exosomes from OLP patients stimulated the production of macrophage inflammatory protein-1 alpha/beta and promoted the migration of CD8+ T cells to the OLP lesions [64]. These exosomes were later found to trigger the apoptosis of keratinocytes in vitro, thus contributing to the exacerbation of OLP [63].

Several studies have suggested the roles of SEs in modulating the gene expression in OLP [15,45,46]. In vitro, fluorescently labeled SEs are taken up by oral keratinocytes and subsequently modulate the oral keratinocytes’ gene expression, by transferring mRNA to the keratinocytes [15]. Besides in vitro studies, the utility of SEs in transporting the miRNAs within humans has also been demonstrated [45]. In 16 OLP patients and 8 healthy controls, Byun et al. revealed that miR-4484 in the SEs derived from OLP patients was significantly upregulated, relative to the healthy controls, indicating that miR-4484 may provide insight into the pathogenic mechanisms of OLP [45].

#### 2.1.2. Periodontitis

Periodontitis is an inflammation of gingival tissue that is characterized by microbial dysbiosis in the oral cavity and a subsequent loss of periodontal ligament attachment and alveolar bone [51,66]. Due to the shared risk factors between periodontitis and various ocular diseases, periodontitis has been shown to be correlated with an increased risk of developing primary open-angle glaucoma [67], cataracts [68,69], scleritis [70], and age-related macular degeneration [71,72]. Non-SEs have exhibited both pro-inflammatory and immunoprotective effects in periodontitis [73,74]. Zhang et al. showed that isolated periodontal ligament stem cell (PDLSCs) exosomes upregulated the inflammation and vascularization of the periodontal ligaments via the transfer of vascular endothelial growth factor (VEGF) [73]. However, the overexpression of exosomal miR-17-5p, an inhibitor of VEGF, slowed the vascularization of the inflamed PDLSCs [73]. Conversely, Shen et al. demonstrated the therapeutic effect of exosomes that were derived from dental pulp stem cells, showing the acceleration of alveolar bone and periodontal epithelium healing in mice with periodontitis [74]. The suppression of periodontal inflammation, by converting the macrophages in the periodontium from pro-inflammatory to anti-inflammatory phenotypes, was also shown [74].

**Figure 2:**
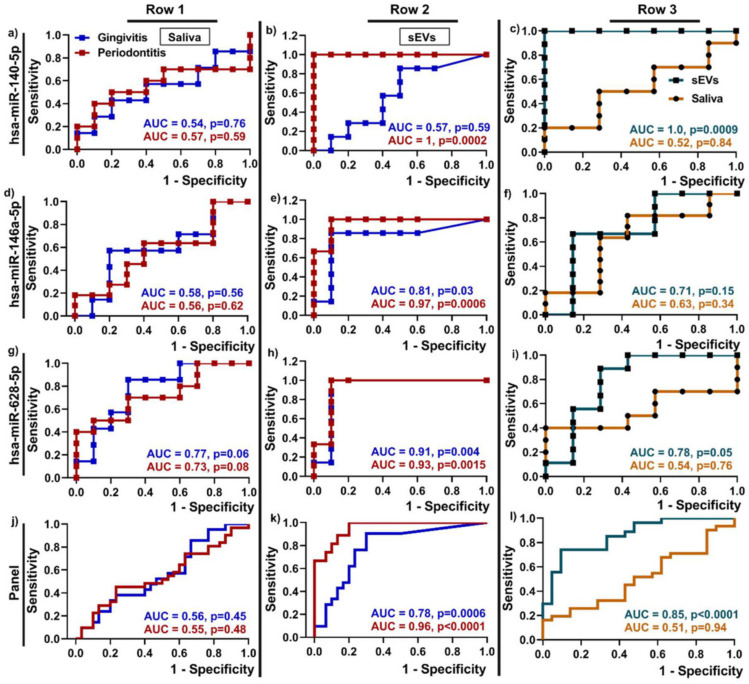
Discriminatory power of upregulated miRNAs hsa-miR-140-5p (**a**–**c**), hsa-miR-146a-5p (**d**–**f**), hsa-miR-628-5p (**g**–**i**), and the miRNA panel (**j**–**l**), in salivary exosomes (SE) from periodontally healthy, gingivitis, and periodontitis patients by using receiver operating characteristics (ROC) curves and area under the curve, AUC. Note: Rows 1 and 2 show discriminatory power between periodontitis or gingivitis vs. periodontal health of whole saliva miRNA (**a**,**d**,**g**,**j**) and SE mRNA (**b**,**e**,**h**,**k**); and Row 3 (**c**,**f**,**i**,**j**) shows the discriminatory power of upregulated miRNAs in saliva and SEs between gingivitis and periodontitis patients [51].

SEs in periodontitis have garnered recent interest [51,75]. Notably, the SEs from periodontitis patients contained higher programmed death-ligand 1 (PD-L1) levels compared to healthy controls, which is positively correlated to the disease severity [75]. Han et al. studied the diagnostic potential of ten miRNAs that were expressed in the SEs, compared to whole saliva that was derived from patients with periodontitis or gingivitis, as well as from healthy individuals [51]. A total of three of those miRNAs (miR-140-5p, miR-146a-5p, and miR-628-5p) were significantly increased in the periodontitis patients, relative to the healthy controls, and were only detected in the SEs from the periodontitis patients, compared to whole saliva, as shown in Figure 2 [51]. When isolated from the whole saliva, miR-140-5p, miR-146a-5p, and miR-628-5p exhibited a low discriminatory power (AUC of 0.57, 0.56, and 0.73, in Figure 2a,d,g) [51]. In contrast, when isolated from the SEs, miR-140-5p, miR-146a-5p, and miR-628-5p demonstrated a high discriminatory power, as indicated by the area under the curve (AUC) of 1, 0.97, and 0.93 (Figure 2b,e,h), highlighting the potential utility of miRNAs in SEs [51].

**Figure 3:**
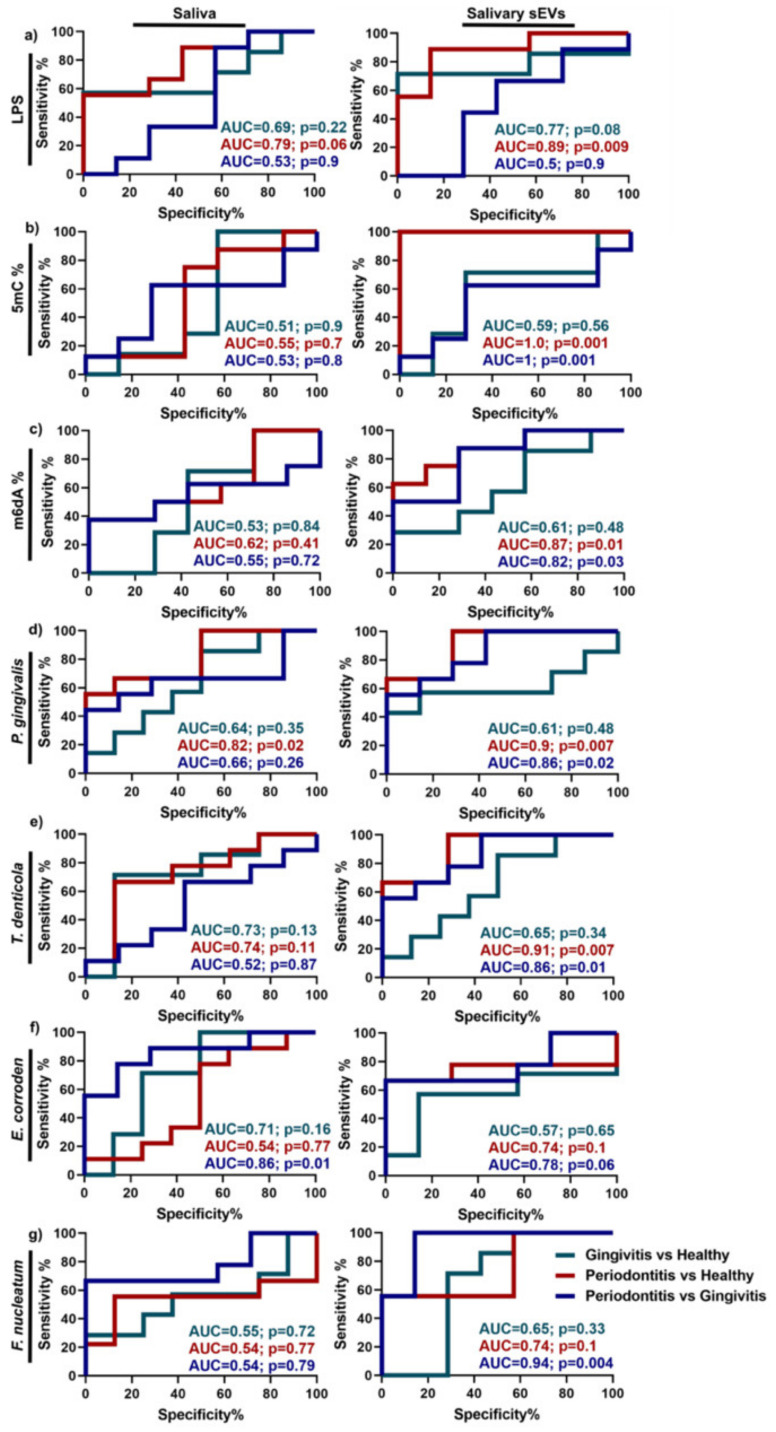
Discrimination power of up-regulated LPS (**a**), global 5mC (**b**), m6dA (**c**), *P. gingivalis* (**d**), *T. denticola* (**e**), *E. corrodens* (**f**), and *F. nucleatum* (**g**) in saliva and salivary SEs from healthy, gingivitis, and periodontitis patients by using receiver operating characteristic (ROC) curves and area under the curve (AUC) [76].

A recent study compared periodontitis patients, gingivitis patients, and healthy individuals by looking at lipopolysaccharide, a virulence factor that is expressed on the outer membrane of Gram-negative bacteria, and the global DNA methylation patterns of the 5-methylcytosine (5mC), 5-hydroxymethylcytosine (5hmC), and N6-Methyladenosine (m6dA) in SEs and whole saliva [76]. The LPS and global 5mC methylation were significantly upregulated in the periodontitis patients compared to the healthy controls, and these differences were more striking in the SEs than in the whole saliva [76]. Additionally, global 5mC hypermethylation could discriminate between periodontitis and gingivitis with a high sensitivity and specificity, as indicated by an AUC of 1 (Figure 3b), suggesting that the global methylation in SEs may be a biomarker for periodontal disease [76]. Together, these data suggest that, compared to whole saliva, SEs represent a source of biomarkers with an improved diagnostic potential for periodontal disease.

#### 2.1.3. Sjogren’s Syndrome

Sjogren’s syndrome (SS) is a chronic autoimmune disorder in which lymphocytic infiltrates trigger the inflammation of exocrine glands [77,78]. The common clinical presentations of SS include keratoconjunctivitis sicca (dry eye) and xerostomia (dry mouth) [77,78]. SS has been associated with an increased risk of vision-threatening ocular morbidities, including optic neuropathy, uveitis, episcleritis, retinal vasculitis, corneal perforation, and cicatrizing conjunctivitis [79]. Since it has been shown that exosomes transport the miRNAs that are involved in autoimmunity and the immune response, the role of exosomes in SS has been explored extensively [52,80,81]. Recent studies have revealed that exosomes that were secreted by T cells derived from SS patients contained higher concentrations of miR-142-3p [81], which has been shown to contribute to the pathogenesis of SS through a decreased cAMP production, calcium signaling, and the subsequent reduced protein production of the salivary glands [81].

Several recent studies have suggested immunoprotective roles for exosomes in SS [52,80]. Using a rabbit model of autoimmune dry eye, Li et al. reported that the exosomes from human umbilical-cord-derived mesenchymal stem cells (MSCs) alleviated ophthalmitis by polarizing peripheral blood macrophages toward the anti-inflammatory M2 phenotype [82]. More recently, the exosomes from labial-gland-derived MSCs were found to enhance the salivary gland function in non-obese mice by decreasing the inflammatory infiltrates in the salivary glands [80]. An intravenous injection of these MSC-derived exosomes stimulated the proliferation of regulatory T cells in SS patients while blocking the production of T-helper 17 (Th17) cells [80]. Similarly, in an in vitro co-culture system of human umbilical cord MSCs and spleen mononuclear cells that were isolated from non-obese diabetic mice, the MSCs downregulated the expression of the pro-inflammatory cytokines (IFN-γ, IL-6, and tumor necrosis factor [TNF]-α) in mononuclear cells [83]. Tested in SS patients, the MSCs induced similar suppressive effects on the T cells, upregulating anti-inflammatory factor IL-10 by nearly three-fold (70 ± 5 pg/mL vs. 18 ± 3 pg/mL) and the regulatory T cells by more than two-fold (6.8 ± 1.6% vs. 3.3 ± 0.6% of total T cells), relative to the healthy controls [83]. Rui et al. demonstrated the therapeutic potential of exosomes that were derived from murine olfactory ecto-b stem cells, which upregulated the immunosuppressive effects of myeloid-derived suppressor cells (MDSCs) and slowed the SS progression, as measured by the increased saliva flow rates and reduced lymphocyte infiltration in the submandibular glands [52]. Specifically, S100A4, a ligand of toll-like receptor (TLR) 4 that is secreted by exosomes, modulated the secretion of IL-6 by MDSCs via TLR4 signaling [52].

Like non-SEs, SEs have been investigated in SS as potential biomarkers and therapeutic targets that can alleviate xerostomia in these patients [20,84,85]. Michael et al. were the first to isolate exosomal miRNAs from the saliva of SS patients [20]. They found that hsa-miR-23a was highly expressed in the SS patients but not in their healthy counterparts, suggesting that the miRNA content of SEs could provide novel markers for the diagnosis of various salivary gland diseases, including SS [20]. Hu et al. found that the expression of 16 peptides and 27 mRNAs was significantly modified in the SS patients compared to the healthy controls [86]. Alevizos et al. also examined the miRNAs that were carried by the SEs and discovered that miR-768-3p and miR-574-3p could identify the SS patients based on their salivary gland focus scores, which rate the intensity of the inflammatory lymphocytic infiltrates [85]. Gallo et al. found that the SEs in SS patients could serve as vehicles for transporting the miRNA that is expressed by the Epstein–Barr virus (ebv-miR-BART13-3p), from B cells to salivary epithelial cells [84].

In addition to miRNA transport, the exosomes from salivary gland epithelial cells carry the autoantigens anti-Ro/SSA, anti-La/SSB, and Sm ribonucleoproteins, suggesting that SEs may mediate the autoantigen presentation of lymphocytes in the progression of SS [87]. Aqrawi et al. used a liquid chromatograph-mass spectrometer (LC-MS) to analyze the proteomic biomarkers in the whole saliva and SEs from SS patients and healthy controls. The study found that the proteins that are involved in innate immunity and wound repair were upregulated in the saliva of the SS patients, when compared to the healthy cohort [88]. Interestingly, neutrophil gelatinase-associated lipocalin, an iron-binding protein that activates neutrophils in the innate immune system, was the most upregulated protein in the SEs of SS patients [88].

#### 2.1.4. Inflammatory Bowel Disease

Inflammatory bowel disease (IBD) is an autoimmune disorder that is characterized by chronic inflammation and ulcers along the colon and rectum [89,90,91]. The common ocular manifestations of IBD include episcleritis [92,93,94,95] and uveitis [94,95,96,97]. Females have been reported to be at a significant risk for developing such ocular manifestations [96,98,99].

The exosomes secreted by MSCs have demonstrated immunoprotective effects in IBD [100,101,102]. For instance, the exosomes from human umbilical cord MSCs have been shown to alleviate the colon tissue inflammation in IBD mice via the upregulation of IL-10 and downregulation of TNF-α, IL-6, and IL-1β [100,103]. The suggested mechanisms for the exosomal protection of colon tissue from damage include suppressing macrophage infiltration into colon tissues [103] and blocking ubiquitination [100], which has been known to contribute to the development of IBD [104,105]. A recent study demonstrated the role of MSC-derived exosomes in inhibiting ubiquitination by transferring miR-326, leading to the downregulation of the pro-inflammatory nuclear factor-kappaB (NF-κB) signaling pathway [106]. More recently, it has been reported that MSC-derived exosomes provided protection against IBD damage by upregulating the TNF-α stimulated gene 6 in mice [101]. In addition, exosomes from human adipose MSCs promoted the regeneration of intestinal stem cells and epithelial cells [102].

Exosomes from sources other than MSCs have also been shown to be able to accelerate and attenuate IBD progression [53,107,108]. Wong et al. used dextran sulfate to simulate acute colitis in a mouse model and examine the role of serum exosomes in the macrophage activation of IBD [107]. The 56 differentially expressed proteins in the exosomes from the acute colitis mice were mainly involved in acute-phase proteins and the immunoglobulins that are involved in the coagulation cascade, which has been associated with macrophage activation [107]. Liao et al. showed that exosomes that were secreted from T regulatory cells relieved IBD by transporting miR-195a-3p to colonic epithelial cells [108]. In contrast, macrophage-derived exosomes that carry miR-21a-5p exacerbate IBD lesions by downregulating the E-cadherin expression and innate lymphoid cell activation in enterocytes [53].

Rather limited research exists on the role of SEs in IBD [47]. In the one and only study that has been reported to date, Zheng et al. extracted SEs from IBD patients and healthy individuals, and more than 2000 proteins were identified using LC-MS [47]. In total, eight of these proteins, which were related to inflammation and proteasome activity, were selected for further analysis [47]. The expression of proteasome subunit alpha type 7 (PSMA7) was significantly elevated in the IBD patients, relative to the healthy controls [47]. PSMA7 is involved in the proteasome activity and inflammatory responses in IBD, and thus can potentially serve as a crucial biomarker for IBD screening [47].

### 2.2. Neurodegenerative Disease

Exosomes from multiple sources have been extensively studied in neurodegenerative diseases [22,48]. In this section, we will discuss Alzheimer’s and Parkinson’s disease, their pathophysiology and ocular manifestations, and the advances in the applications of exosomes [109,110,111], as shown in Table 2.

#### 2.2.1. Alzheimer’s Disease

Alzheimer’s disease (AD) is a neurodegenerative disease that is characterized by progressive cognitive impairment and dementia [112,113,114,115]. The neuropathological lesions in the AD brain include the extracellular deposition of amyloid-beta (Aβ) protein, forming senile plaques, and the hyperphosphorylated tau (p-Tau) protein, forming neurofibrillary tangles [22,112,116,117,118]. The Aβ proteins that are found in the senile plaques of AD are also major constituents of the retinal drusen characteristic of age-macular degeneration, suggesting that AD and AMD share pathogenic mechanisms [119,120]. A significant increase in drusen has been reported in the retinas of AD patients compared to those of healthy patients [121,122]. Multiple studies have also demonstrated a decreased retinal nerve fiber layer thickness in AD patients [123,124,125,126], a decreased contrast sensitivity [127,128,129], and slower, hypometric saccades [130,131,132], when compared to their healthy counterparts. Recently, the hallmark biomarkers of AD, Aβ, and p-Tau have been detected in the retinas of mice models, and methods for the in vivo imaging of these retinal biomarkers require further validation [133,134,135].

It has been shown that exosomes transport Aβ and tau protein, facilitating the propagation of the Aβ and tau pathologies [118,136,137]. Saman et al. observed that neuronal exosomes mediate the secretion of the pathologic tau protein from neurons, reporting tau overexpression in the neuroblastoma cells that recruit mitochondrial proteins, disrupting neural synapses and axons [138]. Higher levels of Aβ and p-Tau proteins have also been detected in the serum exosomes of AD patients, compared to those of healthy controls [139,140]. Goetzl et al. found that, compared to those isolated from the controls, the astrocyte-derived exosomes from AD patients contained significantly higher levels of β-site amyloid precursor protein-cleaving enzyme 1 (BACE-1) and amyloid precursor protein (APP) [137]. Since BACE-1 is required to cleave APP into Aβ peptides [141,142], the astrocyte-derived exosomes that carry BACE-1 represent critical tools for testing the AD therapies that inhibit BACE-1 [137].

Astrocyte-derived exosomes express elevated levels of complement C3 proteins in patients with mild cognitive impairment (MCI) progressing to AD dementia, compared to patients with MCI that remained stable over 3 years [136]. Interestingly, Chiarini et al. showed that exosomes mediate the release of p-Tau from astrocytes, and the levels of p-Tau increased after exposure to Aβ25-35 [143]. Sun et al. studied the urinary exosomes in AD patients for the first time, and reported significantly higher levels of Aβ1-42 and P-S396-tau in the AD patients [144]. Notably, neuronal exosomes were isolated from the induced pluripotent stem cells of a familial AD patient with an A246E mutation to presenilin-1, and injected into the hippocampi of wild-type C57BL/6 mice [145]. Th mice showed increased tau inclusions and an accelerated tau phosphorylation [145]. This study revealed exosomes as a potential mediator of the tau dysregulation that is associated with familial AD [145].

There have been several reports of neuronal exosomes slowing AD progression [109,146]. Yuyama et al. demonstrated the possible neuroprotective roles of neuronal exosomes by facilitating the uptake and clearance of Aβ with microglial cells [147]. The blockade of phosphatidylserine, a surface protein on exosomes, inhibited the exosome-mediated uptake of Aβ into microglia [147]. The inhibition of neutral sphingomyelinase 2 activity reduced the exosome secretion, while the knockdown of sphingomyelin synthase 2 increased the exosome secretion [147]. In addition, the exosomes that are derived from human cerebrospinal fluid (CSF) can sequester Aβ oligomers via surface proteins, such as cellular prion protein [146,148]. Recently, it has been shown that the exosomes from human neural stem cells reduce the Aβ plaque accumulation and microglial activation, protecting against synaptic atrophy [109]. Thus, neuronal exosomes have the potential to mitigate the Aβ-mediated impairment of synaptic plasticity and long-term potentiation [109].

The effects of MSC-derived exosomes on the alleviation of the neuroinflammation in AD is also well described [149,150,151]. MSCs have been observed to slow the memory deterioration of AD mice by increasing anti-inflammatory cytokines, including IL-10, and decreasing the pro-inflammatory cytokines, IL-1β and TNF-α [152]. Exosomes that were isolated from human umbilical cord MSCs were injected into AD mice and found to promote the clearance of Aβ plaques and reduce the levels of pro-inflammatory cytokines [149,153]. Wang et al. showed that exosomes from bone marrow MSCs decreased the Aβ deposition by activating the sphingosine kinase/spingosine-1-phosphate signaling pathway [150], and at the same time, improved spatial learning and cognitive function and reduced the levels of the BACE1, Aβ1-40, and Aβ1-42 peptides [150]. Since BACE1 cleaves the amyloid precursor protein to form the Aβ1-40 and Aβ1-42 peptides, reducing the BACE1 levels is critical to slowing the Aβ accumulation and disease progression [142,154]. Using a neural cell culture model that simulated AD by overexpressing APP, Chen et al., demonstrated that mesenchymal stem cell-derived exosomes reduced Aβ production [151]. Exosomal miR-29a enhanced the expression of the genes that are involved in synaptic plasticity, by downregulating the expression of histone deacetylase 4, which is markedly increased in AD patients [151,155].

While studies on the exosomes in AD have been plenty, SEs specifically have only recently been examined [22]. Rani et al. used a nanoparticle tracking analysis to quantify and analyze the SEs in 10 cognitively impaired patients, 5 AD patients, and 12 healthy controls [22]. The total concentration of the SEs was significantly higher in the cognitively impaired and AD patients, when compared to the healthy controls [22]. Furthermore, the elevated levels of SEs were correlated with a decrease in the ACEIII value, indicating an increased cognitive impairment and disease severity [22]. These results confirmed that the oligomeric forms of the Aβ protein and p-Tau protein were significantly higher in the cognitively impaired and Alzheimer’s disease patients [22]. Together, these findings demonstrate the utility of a nanoparticle tracking analysis to quantify the SEs’ concentration, and to serve as a cost-efficient screening method for the early detection of AD [22].

#### 2.2.2. Parkinson’s Disease

Parkinson’s disease (PD) is a movement disorder that occurs due to the degeneration of the pigmented neurons in the substantia nigra, involving Lewy bodies that contain α-synuclein protein [48,156,157,158]. Its hallmark clinical features include bradykinesia (slowness of movement), resting tremors, postural disequilibrium, and ataxia [158,159]. The bradykinesia manifests not only in the limbs, but also in the eyes, as impaired smooth pursuit [160,161]. Other oculomotor abnormalities in PD include an impaired convergence [162,163,164,165], diplopia [165,166,167], and an impaired vertical gaze [160,168]. PD has also been associated with a higher incidence of dry eye [164,169,170,171] and glaucoma [172,173].

PD-derived exosomes can trigger the formation of α-synuclein oligomers, which are then packaged in exosomes for disease propagation [174]. In addition, evidence of α-synuclein transport via the exosomes from CSF to the venous circulation implicates the role of these exosomes in PD progression [175,176]. Stuendl et al. showed that the exosomes from the CSF of PD patients carried higher levels of α-synuclein, and suggested that CSF exosomes may disseminate the PD pathology by triggering the oligomerization of α-synuclein [177]. Similarly, treatment with human α-synuclein preformed fibrils induced the microglia to release exosomes containing α-synuclein [178]. By degrading the lysosomal structural protein LAMP2, the α-synuclein preformed fibrils also impaired the lysosomal breakdown of exosomal α-synuclein from microglia, indicating that microglial-derived exosomes contribute to the α-synuclein aggregation in neurons [178,179,180]. Recently, Si et al., reported that the exosome secretion by microglial cells is increased by the NOD-like receptor family pyrin domain, containing three protein (NLRP3) inflammasome, which is known to be involved in the neuroinflammation of PD [181].

Elevated α-synuclein levels have also been found in plasma neural-derived exosomes [182,183]. After the plasma exosomes from PD patients were injected into mice brains, the microglia showed an increased uptake of these exosomes and the release of exosomal α-synuclein, implying that microglia may facilitate the transmission of exosomal α-synuclein [184]. The plasma exosomes were also found to dysregulate the autophagy in mouse microglia, as measured by the increased accumulation of α-synuclein in the intracellular and extracellular spaces [111,184]. Besides α-synuclein, serum exosomes have been shown to carry other biomarkers of PD [185,186]. For example, miR-137, which is known to induce the oxidative stress of neurons in PD [186], was detected in increased levels in the serum exosomes of PD patients, and was shown to downregulate oxidation resistance 1 (OXR1) [185]. The depletion of miR-137 or the upregulation of OXR1, on the other hand, alleviated PD-induced oxidative stress [185].

The neuroprotective effects of exosomes in PDs have also been reported [110,187,188]. The treatment of PD mice with blood-derived exosomes from healthy human subjects was found to reduce the loss of dopaminergic neurons, alleviate oxidative stress and inflammation, and improve the motor coordination in PD mice [188]. In addition, this exosome treatment significantly reduced the mRNA expression of the pro-inflammatory cytokines, IL-1beta, IL-6, and TNF- a, and increased the mRNA levels of the anti-inflammatory cytokines IL-4, IL-10, and TGFB, indicating that exosomes can attenuate neuroinflammation [188]. The exosomes secreted by human umbilical cord MSCs demonstrated a neuroprotective role in PDs [110], by crossing the blood–brain barrier to reduce the apoptosis of the dopaminergic neurons in the substantia nigra [110]. Specifically, miR-188-3p, which is carried by MSC exosomes, suppressed neuron loss by targeting the NLRP3 autophagy pathway [187]. The treatment with MSC-derived exosomes also promoted the proliferation of neuronal cells that were subjected to 6-hydroxydopamine (6-OHDA)-mediated oxidative stress [110]. These findings illustrate the protective ability of MSC exosomes in PD.

Although limited, there is evidence for SEs having a role in PD propagation [48,189]. In a study of 74 PD patients and 60 healthy controls, Cao et al. reported significantly higher levels of oligomeric α-synuclein and higher ratios of oligomeric α-synuclein to the total α-synuclein in the SEs from PD patients, relative to the healthy controls [48]. This was supported by another study of 18 PD patients and 15 healthy controls that found the ratio of hyperphosphorylated α-synuclein to the total α-synuclein to be significantly higher in the PD-derived SEs [189]. These data support the idea that the pathogenic mechanism of PD involves SEs disseminating the misfolded α-synuclein proteins in the brain [48,189].

### 2.3. Malignant Neoplasms

Exosomes have been used to identify the markers for tumor migration, proliferation, and metastasis [190,191]. It has been found that exosomes contain miRNA and circulating tumor genes (ctDNA), which are both involved in intercellular communication in the tumor microenvironment [192,193,194]. In this section, we will discuss various cancers, their ocular effects, and the roles of exosomes in cancer development and progression. A summary of the cancers, exosomal biomarkers, and their effects, are shown in Table 2.

#### 2.3.1. Oral Cancers

Oral cancers, also known as head and neck cancers, are malignancies that often occur in the oral cavity, especially the tongue and the floor of the mouth, the oropharynx, and the esophagus [195,196,197,198]. Oral squamous cell carcinoma (OSCC) is the most common type of oral cancer in the world [199]. The risk factors that are involved in the development of oral cancer in Western countries include the male sex and an age of over 65 years [195,197,199]. Its etiologic agents include smoking and alcohol consumption [195,199,200]. While the risk factors and etiology of oral cancers have been extensively studied, little is known about the ocular effects of oral cancers [201,202,203]. In one of the few studies that have been reported, an increased incidence of dry eye disease, as indicated by a reduced tear film break up time and a decreased tear production, has been found in oral cancer patients [201]. Few cases of the ocular metastases of oral cancer have been reported, occurring through choroidal spread [202,203].

Although limited, the findings on exosomes in oral cancers have demonstrated that they can both inhibit and promote cancer cell growth [49,50,204]. Human bone marrow MSC-derived exosomes that overexpress miR-101-3p inhibit oral cancer progression by downregulating COL10A1 [204]. However, plasma-derived exosomes showed immunosuppressive effects that facilitate the progression of head and neck squamous cell carcinomas (HNSCC) and esophageal cancer [50]. For example, miR-93-5p and miR-19b-3p, transferred by plasma exosomes, promote the proliferation of esophageal cancer cells by inhibiting PTEN expression [49,205]. Furthermore, exosomes from the plasma of HNSCC patients with active disease, compared to the plasma exosomes from patients with no evident disease, more effectively inhibited CD4(+) T-cell proliferation, promoted the apoptosis of CD8(+) T cells, and increased the regulatory T-cell suppressor functions [206]. Notably, PD-L1, a known tumorigenic factor that is carried by the plasma exosomes from HNSCC patients, is linked to disease progression, as the blocking of PD-L1 signaling attenuates immune suppression [50].

The literature on SEs in relation to oral cancers is more prolific [207,208,209]. Wang et al. investigated the potential of human papillomavirus DNA that was extracted from human saliva as a biomarker for HNSCC. The authors observed that the tumor DNA that was detected in all the early stage disease patients (*n* = 10), and in 95% of the late-stage disease patients (*n* = 37), was significantly higher than the proportions of the tumor DNA that was found in the plasma of the HNSCC patients [208]. To examine the differences in the exosomal miRNA of HNSCC patients, relative to the controls, Langevin et al. cultured four discrete HNSCC cell lines, originating from four different sites in the upper digestive tract: H413 (buccal mucosa), Detroit 562 (pharynx), FaDu (hypopharynx), and Cal 27 (tongue), as well as human gingival epithelial cells from healthy donors for comparison [207]. Higher levels of miR-486-5p, miR-486-3p, and miR-10b-5p were detected in the HNSCC patients relative to the controls [207]. Importantly, miR-486-5p was also found in the early-stage lesions, implicating its role in early disease detection [207]. Furthermore, miR-302b-3p and miR-517b-3p were expressed in the SEs from oral squamous cell carcinoma (OSCC) patients, and miR-512-3p and miR-412-3p were higher in the OSCC patients [209]. Elevated levels of GOLM1-NAA35 chimeric RNA were reported in esophageal squamous cell carcinoma tissues [210]. Other studies on OSCC patients and healthy controls demonstrated that miR-29a-3p promoted tumor growth by inducing M2 subtype macrophage polarization via the SOCS1/STAT6 signaling pathway [211]. An increased expression of miR-29a-3p [211] and miR-31 [212] was found in the exosomes of OSCC patients. These exosomal miRNA levels were significantly decreased after a tumor resection, indicating their utility in monitoring cancer progression [212].

#### 2.3.2. Breast Cancer

Breast cancer is the most common type of cancer affecting women worldwide [213,214,215]. Although rare, the ocular metastases of breast cancer have been reported in both female and male patients [216]. The breast is a common origin site of ocular metastatic tumors, accounting for 49% of patients [216]. Since most ocular metastases occur through hematogenous dissemination, the uveal tract, especially the choroid, due to its vascularity, is the most common site of origin for breast cancer metastases [217,218]. A common complication of metastatic choroidal disease in breast cancer patients is vision deterioration, due to a macular invasion of the tumor or fluid accumulation in the fovea [219,220].

Exosome studies of breast cancer have gained recent traction [221,222]. Sun et al. demonstrated that RAB22A, a proto-oncogene involved in the production, trafficking, and metabolism of exosomes, upregulates exosome-mediated breast cancer cell proliferation, invasion, and migration [223]. However, miR-193b downregulates the oncogenic effects of RAB22A, hindering exosome-induced cancer cell metastasis [223]. Additionally, oncogenic miRNAs, miR-21 and miR-200c, have been detected in higher levels in the tear exosomes of breast cancer patients compared to healthy subjects [224]. Notably, miR-21 was increased by nearly 3-fold and miR-200c was increased by 15-fold, suggesting that these tear exosomes can be a useful source of diagnostic and prognostic markers for breast cancer [224]. Ando et al. found that the matrix metalloproteinase-1 (MMP-1) in the urinary exosomes from breast cancer patients was significantly elevated compared to the healthy controls [225]. In contrast, the miR-21 expression in the patients was significantly lower than in the controls, indicating that MMP-1 and miR-21 may be potential screening markers for breast cancer [225]. Shtam et al. reported that the plasma exosomes from healthy donors facilitated the migration and transwell invasion of breast cancer cells, and promoted their metastatic spread [221]. This effect was mediated by the interactions of exosome surface proteins with breast cancer cells, stimulating focal adhesion kinase signaling in the breast cancer cells [221]. Shtam et al. concluded that plasma exosomes have the potential to trigger the metastasis of breast cancer cells [221].

While other types of exosomes, including those from plasma, tears, and urine, have been shown to induce breast cancer progression, some studies have demonstrated the therapeutic effects of MSCs in breast cancer [222,226,227,228,229]. MSC-derived exosomes inhibit the VEGF expression and angiogenesis of breast cancer cells, by transporting miRNA-100 via the mTOR/hypoxia-inducible factor (HIF)-1-α signaling pathway [226,227]. Furthermore, the human umbilical cord MSC-derived exosomes that carry miR-148b-3p inhibited the breast cancer cell proliferation, migration, and invasion [228]. Recently, exosomes from adipose MSCs inhibited breast cancer metastasis and epithelial-to-mesenchymal transition (EMT) via the downregulation of DNA repair genes (PARP1 and CCND2) and cancer stem cell surface markers (CD44 and ALDH1) [229]. Similarly, these adipose MSC-derived exosomes, loaded with miR-381, significantly reduced the expression of EMT-related genes, inhibiting the proliferation, migration, and invasion of breast cancer cells [222].

There have been several studies of SEs in breast cancer [230,231]. Zhang et al. reported higher levels of carbonic anhydrase 6 (CA6) and insulin-like growth factor 2 mRNA-binding protein 1 (IGF2BP1) in the saliva of breast cancer patients compared to controls [232]. Notably, elevated levels of CA6 and CA15-3 have been detected in the saliva of breast cancer patients [233,234]. Similarly, TCTP1 and IGF2BP1 promote the proliferation of various cancers, including breast cancer, and TCTP1 overexpression stimulates the degradation of the tumor suppressor gene, p53 [230,235]. Lau et al. treated salivary gland exosomes with breast-cancer-derived exosomes to examine the interactions between them [231]. The authors detected 88 proteins in the treated salivary gland exosomes at levels that were 1.5 times higher than those in the control group, and 66 mRNAs that were differentially expressed by the treated exosomes, indicating the ability of breast-cancer-derived exosomes to modify the content of both the proteins and mRNAs in SEs [231]. The breast-cancer-derived exosomes also upregulated the total RNA that was expressed by the salivary gland exosomes [231]. This increase in the total RNA was significantly decreased after the salivary glands were exposed to the transcription inhibitor actinomycin D [231]. These results suggest that the upregulated RNA transcription that was detected in the SEs was mediated by the interplay between the salivary gland exosomes and breast-cancer-derived exosomes [231].

#### 2.3.3. Colorectal Cancer

Colorectal cancer is the third most common cancer in men and second most common cancer in women worldwide [213]. Colorectal cancer is the second cause of cancer-related deaths in Western countries, accounting for 9% of all cancer-related deaths [236]. While rare, several cases of intraocular metastasis from colorectal cancer exist, disseminating via the choroid [237,238].

Although scarce, reports on the exosomal markers for colorectal cancer exist [239,240,241]. Xiao et al. reported that cytokeratin 19 (CK19) was enriched in the exosomes that were derived from colorectal cancer cells, that carbohydrate antigen 125 (CA125) was elevated in the exosomes from metastatic colorectal cancer cells, and that tumor-associated glycoprotein 72 (TAG72) was detected in the exosomes from 5-fluorouracil (5-FU)-resistant colorectal cancer cells [240]. CK19, CA125, and TAG72 were also found in the interstitial-fluid-derived exosomes and serum-derived exosomes of colorectal cancer patients [240]. Additionally, Li et al. found a higher concentration of circular RNAs (circRNAs) that were secreted by the serum exosomes from colorectal cancer patients, supporting the utility of circRNAs in cancer diagnosis [241]. Recently, angiopoietin-like protein 1 (ANGPTL1), which was detected in exosomes that were derived from human colorectal cancer cells, has been shown to inhibit the liver metastasis of colorectal cancer cells by blocking MMP9-induced vascular leakiness via the JAK2-STAT3 pathway [239].

The SEs in colorectal cancer have attracted recent interest, but much is still unknown about their function in its pathogenesis [242,243]. Sazanov et al. detected, using a qRT-PCR, a significantly higher miR-21 expression in the SEs from colorectal cancer patients compared to controls, and this miR-21 expression demonstrated a high diagnostic sensitivity and specificity (97% and 91%, respectively) [242]. Other salivary miRNAs, including miR-186-5p, miR-29a-3p, miR-29c-3p, miR-766-3p, and miR-491-5p, were significantly elevated in colorectal cancer patients [243]. Together, the literature suggests that SEs may not only represent a critical tumor screening tool, but may also be used to treat malignant tumors, by impeding tumor invasion and growth [242,243].

#### 2.3.4. Lung Cancer

Lung cancer is the leading cause of cancer mortality in men and women in the United States, accounting for nearly 25% of cancer deaths [213]. Smoking is the number one risk factor for developing and dying from lung cancer [213]. Like most ocular metastases from solid tumors, orbital involvement from primary lung cancer most commonly affects the choroid [244,245,246]. The prognosis of lung cancer with choroidal metastasis is poor, with a median survival of 6-13 months [247]. There have also been a few rare cases of iris metastases, secondary to lung tumors [248,249,250].

Exosomal applications in lung cancer have gained attention in recent years. Rahman et al. observed that exosomes from metastatic lung cancer cells induced vimentin expression and EMT, as well as migration, invasion, and proliferation in non-cancerous human bronchial epithelial cells [251]. The authors concluded that lung-cancer-derived exosomes could drive healthy recipient cells towards EMT [251]. Xiao et al. found that the exosomes secreted by A549 lung cancer cells decreased the sensitivity of the A549 cells to cisplatin, and the increase in chemoresistance may be mediated by the exosomal transport of miRNAs [252]. More recently, exosomes from lung cancer bronchoalveolar lavage fluid were found to promote the migration and invasion of A549 lung cancer cells by carrying E-cadherin [253]. Macrophage-derived exosomes have also been shown to promote cisplatin resistance in lung cancer [254]. By delivering miR-3679-5p to A549 lung cancer cells, macrophage-derived exosomes enhance aerobic glycolysis and chemoresistance, via the NEDD4L/c-Myc signaling cascade [254].

**Table 2:** Exosomes in non-ocular diseases.

Disease	Exosome Type	Biomarkers	Effect	Reference
**Alzheimer’s Disease**	**Saliva**	Aβ, p-Tau Aβ1-42, P-S396-tau	Disease propagation via Aβ and tau deposition	[14]
	**Blood**			[131,132]
	**Urine**			[136]
	**Astrocytes**	BACE-1, sAPPβ, Complement C3, p-Tau		[129]
				[128]
				[135]
	**CSF**	Cellular prion protein	Neuroprotective; reduce exosome uptake of Aβ	[140]
	**MSCs**	BACE1, Aβ1-40, Aβ1-42	Inhibit Aβ deposition	[142]
		miR-29a	Increase synaptic plasticity via HDAC4 downregulation	[143,147]
**Parkinson’s Disease**	**Saliva**	α-synuclein oligomers	Disease propagation via α-synuclein oligomerization	[40,181]
	**CSF**			[169]
	**Microglia**		Inhibit lysosomal breakdown of α-synuclein	[170]
	**Serum**		Uptake of α-synuclein into microglia	[176]
		miR-137	Increase oxidative stress via OXR1 inhibition	[177]
**Oral Lichen Planus**	**Saliva**	miR-4484, miR-146a, miR-155	Unknown	[37,38]
	**T cells**	MIP-1 alpha/beta	T-cell migration; apoptosis of keratinocytes	[55,56]
**Periodontitis**	**Saliva**	miR-140-5p, miR-146a-5p, miR-628-5p LPS, 5mC methylation	Unknown	[43,68]
	**Periodontal ligament stem cells**	miR-17-5p	Inhibit inflammation and angiogenesis	[65]
**Sjorgen’s Syndrome**	**Saliva**	miR-768-3p, miR-574-3p	Unknown	[77]
		Anti-Ro/SSA, anti-La/SSB, Sm ribonucleoproteins	Autoantigen presentation to lymphocytes	[79]
		CD44 antigen; NGAL	T cell activation; neutrophil activation	[80]
	**T cells**	miR-142-3p	Decrease protein production from salivary glands via cAMP inhibition	[73]
**Inflammatory Bowel Disease**	**Saliva**	PSMA7	Proteasome activity and inflammatory response	[39]
	**MSCs**	miR-326	Inhibit ubiquitination via NF-kappaB downregulation	[98]
	**T regulatory cells**	miR-195a-3p	Reduce inflammation	[100]
	**Macrophages**	miR-21a-5p	Exacerbate IBD via E-cadherin inhibition	[45]
**Oral squamous cell carcinoma**	**Saliva**	miR-302b-3p, miR-517b-3p, miR-512-3p, miR-412-3p	Unknown	[201]
		miR-31, miR-29a-3p	Promote M2 subtype macrophage polarization	[203,204]
	**MSCs**	miR-101-3p	Inhibit cancer progression via COL10A1 downregulation	[196]
**Esophageal Cancer**	**Saliva**	GOLM1-NAA35 chimeric RNA	Unknown	[202]
	**Plasma**	miR-93-5p; miR-19b-3p	Proliferation of esophageal cancer cells via PTEN inhibition	[41,197]
**Head and Neck**	**Saliva**	Human papillomavirus DNA, miR-486-5p, miR-486-3p, miR-10b-5p	Unknown	[199,200]
**Breast cancer**	**Saliva**	CA6, CSTA, TPT1, IGF2BP1	Unknown	[224]
	**Urine**	MMP-1	Pro-angiogenic	[217]
	**MSCs**	miRNA-100, miR-148b-3p, miR-381	Inhibit angiogenesis and cancer cell proliferation	[214,218,220]
**Colorectal cancer**	**Saliva**	ANGPTL1	Blocks metastasis via MMP9 inhibition	[238]
		miR-21, miR-186-5p, miR-29a-3p, miR-29c-3p, miR-766-3p, miR-491-5p	Unknown	[241,242]
**Lung cancer**	**Saliva**	BPIFA1, CRNN, MuC5B, IQGAP	Unknown	[254]
	**Lung cancer bronchoalveolar lavage fluid**	E-cadherin	Promote cancer cell migration and invasion	[252]
	**Macrophage**	miR-3679-5p	Promote aerobic glycolysis and chemoresistance	[253]

The literature on using SEs and lung cancer mechanisms is limited. In one of the few studies that have been reported, Sun et al. compared the protein composition of the SEs in lung cancer patients with that of normal subjects, and verified the presence of four proteins that are specific to lung cancer tissue (BPIFA1, CRNN, MuC5B, and IQGAP) [255].

## 3. Exosomes in Ocular Diseases

In addition to non-ocular diseases, exosomes have been implicated in the development and/or progression of several ocular diseases [256,257,258,259]. The subsequent sections will highlight the roles of exosomes in diabetic retinopathy, corneal disease, age-related macular degeneration, uveal disease, and glaucoma. Table 3 summarizes the ocular diseases, exosome biomarkers, and their effects.

### 3.1. Diabetic Retinopathy

Diabetic retinopathy (DR) is a complication of diabetes that is caused by damage to the blood vessels in the retina [260,261]. DR remains the leading cause of blindness and visual impairment in working-aged adults and is a growing public health problem [261,262]. The global diabetes prevalence in 2021 was estimated to be 537 million (10.5%), and this figure is projected to increase to 783 million by 2040 (12.2%) [263].

Non-proliferative DR is the early stage of the disease, in which the blood vessels in the retina are weakened, leading to the formation of microaneurysms and the swelling of the macula [260,264]. The early histological features of DR include retinal endothelial cell loss and the thickening of the retinal vascular basement membrane, correlating with increased fibronectin levels [265,266,267]. Proliferative DR is the more advanced form of the disease, marked by retinal hypoxia and resulting in neovascularization, in which new, fragile blood vessels begin to grow in the retina and into the vitreous [268]. If left untreated, PDR can cause retinal detachment, severe vision loss, and blindness [268,269,270,271]. Since the pathogenesis of proliferative DR has been shown to involve the neovascularization of retinal cells, exosomes contribute to the disease development by regulating the angiogenic factors [272,273]. For example, Tokarz et al. found that, relative to the exosomes of healthy controls, the exosomes of diabetic patients carried significantly higher pro-inflammatory and pro-angiogenic factors, such as basic fibroblast growth factor, TNF-α, VEGF receptor 2, and angiopoietin-2 [273]. More recently, Maisto et al. exposed retinal photoreceptors to high glucose concentrations (30 mM) and found a significant upregulation of VEGF and downregulation of anti-angiogenic miRNAs, including miR-20a-3p, miR-20a-5p, and miR-20b, in the exosomes that were released by the retinal cells [272].

Plasma exosomes have been shown to carry critical biomarkers and induce oxidative damage in DR [274,275,276]. Specifically, plasma exosomes carry elevated amounts of peroxisome proliferator-activated receptor gamma (PPAR-γ) in the aqueous humor and vitreous fluid of proliferative DR patients, relative to healthy controls [277]. A significant positive correlation between the PPAR-γ and VEGF concentrations was also found, together with notable increase in the PPAR-γ levels, which was correlated with the clinical progression of DR, delineating the role of PPAR-γ in the pathogenesis of DR [277]. Huang et al. discovered that plasma exosomes also contain IgG, which activates the classical complement pathway and inflammatory responses, and showed that these exosomes are increased in DR [278]. Huang et al. later found that IgG-carrying exosomes cause damage to the retinal endothelial cells by initiating membrane attack complex (MAC) formation and activating the complement system [274]. Zhang et al. similarly noted that exosomes from platelet-rich plasma (PRP-Exos) exacerbated hyperglycemia-induced retinal ischemia [275]. PRP-Exos were upregulated in diabetic rats via the TLR4 signaling pathway, mediating the inflammation of the retinal endothelial cells [275].

Multiple studies have implicated the therapeutic potential of exosomes in diabetic retinopathy [279,280,281]. Using an oxygen-induced retinopathy mouse model, Moisseiev et al. investigated the effect of exosomes on retinal ischemia, by intravitreally administering exosomes that were derived from human MSCs [282]. Retinal thinning and angiogenesis were significantly reduced in the ischemic retinas that were treated with the MSC-derived exosomes compared to the eyes that were treated with saline [282]. A similar therapeutic role of these MSC-derived exosomes was exhibited in a rabbit model of DR [281]. The administration of the MSC-derived exosomes from rabbit adipose tissue caused the regeneration of the normal retinal layers and increased the expression of miRNA-222, indicating that exosomes may induce retinal repair via the miRNA-222 transfer to damaged cells [281].

Like MSC-derived exosomes, exosomes from retinal cells have also been implicated in the retinal repair of DR lesions [279,280,283]. For instance, Liu et al. demonstrated that exosomes carry circular RNA-cPWWP2A, which indirectly modulates endothelial cell activity via the inhibition of miR-579 [283]. The upregulation of cPWWP2A expression, due to diabetes-induced stress, was shown to reduce retinal endothelial damage [283]. Another study, a year later, found that the retinal exosome depletion by GW4869 impeded the transport of photoreceptor-derived miR-124-3p to the inner retina [279]. Exosomal inhibition by GW4869 also aggravated the retinal lesions and induced further inflammation and damage to the photoreceptors, implying a role of retinal exosomes in protecting against retinal degeneration [279]. Interestingly, Gu et al. suggested that retinal pigment epithelium (RPE)-derived exosomes suppress the pathologic fibrosis in proliferative DR by transferring miR-202-5p to human umbilical vein endothelial cells (HUVECs). miR-202-5p, which is carried by exosomes, negatively regulated TGF-β2, resulting in the inhibition of the HUVEC proliferation and tube formation [280].

### 3.2. Retinitis Pigmentosa

Retinitis pigmentosa is a retinal degenerative disease, in which rod photoreceptors are lost due to inherited gene mutations [284,285]. While this loss of rods leads to night blindness, the progression of the disease may cause total vision loss when the cone photoreceptors are irreversibly damaged [284]. Previous studies have demonstrated that poly-ADP-ribose polymerase (PARP) hyperactivity may be involved in the retinal degenerative process of retinitis pigmentosa [286,287]. Vidal-Gil et al. examined the relationship between PARP inhibition and exosome secretion [288]. The authors found that CD9, a tetraspanin protein on the surface of exosomes, was in the outer nuclear layer (ONL) and inner retina, suggesting that a significant proportion of exosomes may be secreted from retinal photoreceptors [288]. Furthermore, after the inhibition of the PARP activity, the CD9 expression was reduced in the ONL and inner layers of the retina, while the photoreceptor degeneration was significantly improved [288]. Thus, decreasing the secretion of CD9-expressing retinal exosomes might help to alleviate photoreceptor damage [288].

### 3.3. Age-Related Macular Degeneration

Age-related macular degeneration (AMD) is a leading cause of central vision loss among the elderly [289]. The pathogenesis of the early, non-exudative, “dry” type of AMD involves the formation of drusen, a lipid-like material that deposits on Bruch’s membrane in the macula of the retina [290,291,292,293]. These drusen deposits disrupt the retinal blood supply, resulting in a loss of photoreceptors and vision loss [291,292,293,294]. The late, exudative, “wet” type of AMD is characterized by macular neovascularization and retinal endothelial leakage, leading to severe, rapid vision loss [291,293,295,296].

Mutations in complement factor H and the dysfunction of complement protein C3 are associated with an increased risk for the development of AMD [297,298]. Wang et al. observed that exosomes released by the RPE of AMD patients are coated with C3 and can interact with complement factor H (CFH), and proposed that the mutated CFH in AMD may disrupt the exosomal release of proteins and the subsequent incorporation of these proteins into drusen [290]. The proteins CD63, CD81, and LAMP2 were also detected in the RPE-derived exosomes and drusen of AMD patients, but not in the healthy controls, providing evidence that RPE-derived exosomes contain proteins that promote drusen production [290]. Kang et al. identified the exosomal proteins secreted by RPE that may serve as potential biomarkers for AMD [299]. Specifically, higher levels of cathepsin D and cytokeratins 8 and 14 were found in the aqueous-humor-derived exosomes of AMD patients [299]. The authors concluded that the upregulation of these proteins may be due to a reactive response against the oxidative stress mediated by the autophagy-lysosomal pathway [299].

Several studies have suggested that the macular neovascularization and retinal endothelial leakage of exudative AMD may be mediated by the RPE-derived exosomes that express VEGF receptors [300,301]. A recent in vitro assay found that the serum-derived exosomes from AMD patients were upregulated in miR-19a, miR-126, and miR-410, and formed significantly more endothelial tubules, relative to the serum-derived exosomes from controls [302]. Bioinformatics analyses confirmed that these exosomal miRNAs were associated with the VEGF/angiogenesis and apoptosis signaling pathways [302]. These findings demonstrated a pathogenic role of serum exosomes in AMD by promoting the neovascularization and apoptosis of retinal cells [302]. miR-126, miR-410, and miR-19a have been previously implicated in retinal disease [303,304,305]. miR-126 promotes VEGF expression and retinal neovascularization, while miR-410 suppresses VEGF-mediated neovascularization [303,304,305]. Interestingly, increasing the miR-19a levels in RGCs has been shown to promote axon regeneration in vivo after an optic nerve crush in mice, and in RGCs from human donors [306].

While the exosomes from serum and RPE exhibit pro-angiogenic effects, the exosomes that are released from retinal astroglial cells (RACs) have anti-angiogenic properties [307]. In a model of laser-induced choroidal neovascularization (CNV), RAC-derived exosomes inhibited CNV and vascular lesions, while the exosomes that were secreted by RPE did not [307]. Anti-angiogenic agents, such as endostatin and MMP3, were detected in the RAC-derived exosomes [307]. These findings indicated that the exosomes that are released from RACs downregulate angiogenesis by suppressing the migration of macrophages and vascular endothelial cells, both of which have been associated with the development of exudative AMD [307]. More recently, it has been reported that exosomes that are released from mouse neural progenitor cells (NPCs) have therapeutic effects in AMD, by slowing retinal degeneration [256], exhibiting inhibitory effects on microglial cells, and mitigating photoreceptor apoptosis and the atrophy of the ONL [256].

### 3.4. Corneal Diseases

The role of exosomes in corneal wound healing has been examined in various studies [308,309,310,311]. Corneal fibroblasts have been shown to secrete exosomes that deliver MMP14 and other angiogenic proteins to endothelial cells, and MMP14 is involved in the incorporation of MMP2 into corneal fibroblast exosomes [311]. These findings suggest that the MMP14 in exosomes may be a key therapeutic target for angiogenesis and the mediation of the cell–cell communication in the cornea [311]. The same group of investigators also found that the exosomes that are secreted by mouse corneal epithelial cells, upon fusing to target keratocytes, stimulated the differentiation of myofibroblasts and the expression of alpha-smooth muscle actin (α-SMA), a known marker of fibrosis [310]. As myofibroblast differentiation is implicated in corneal wound closure and neovascularization, corneal epithelial-derived exosomes may be important therapeutic targets for corneal disease [310]. Leszczynska et al. found that exosomes from limbal stromal cells (LSCs) accelerated the limbal epithelial stem cell (LESC) proliferation and wound healing via Akt phosphorylation and the upregulation of LESC markers, including keratin 15 [312].

**Figure 4:**
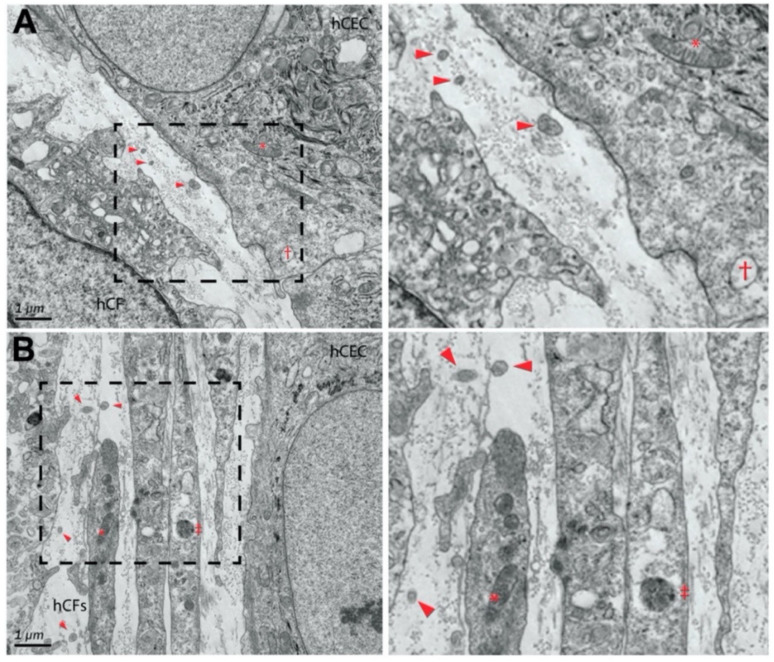
TEM of corneal epithelial–stromal interactions. (**A**) The presence of secreted collagen and extracellular vesicles were apparent between human corneal epithelial cells (hCECs) and human corneal fibroblasts (hCFs) (dashed box enlarged in right panel). (**B**) Extracellular vesicles were also present in between hCF cell populations (dashed box enlarged in right panel). Arrowheads = extracellular vesicles; * = mitochondria; † = vacuole; and ‡ = lysosome. Magnification = 12,000× [313].

**Figure 5:**
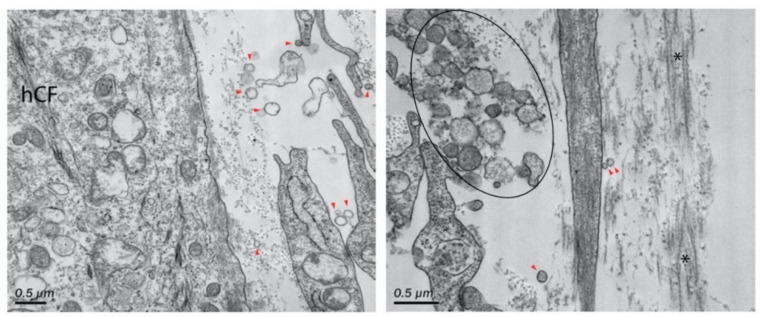
TEM of corneal stromal interactions at high magnification. Secreted extracellular vesicles were identified within the stroma (red arrowheads). Large aggregates of extracellular vesicles (50–330 nm) were also found within the stroma near adjacent cells (black ellipse). Asterisks (*) denote deposited collagen fibrils. Magnification = 21,000× (left panel) and 31,000× (right panel) [313].

**Figure 6:**
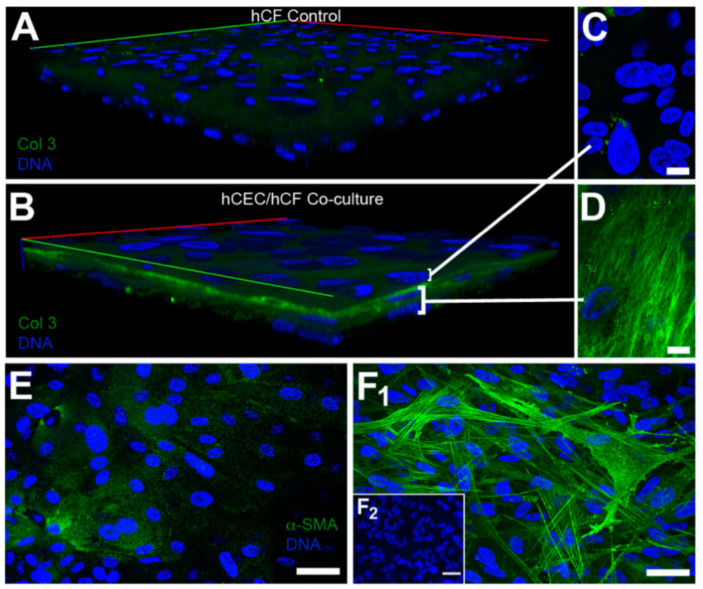
Expression of fibrotic markers in stromal constructs and epithelial–stromal co-cultures. Expression of collagen type III (Col 3) in (**A**) hCF control constructs, and (**B**) epithelial–stromal (hCEC/hCF) co-culture. High magnification of a single plane of focus in the (**C**) epithelial layer, and (**D**) stromal layer, show high expression of collagen type III by hCFs and little expression by hCECs. Expression of α-smooth muscle actin in the (**E**) epithelial layer (max projection), and (**F_1_**) stromal layer (max projection), of a co-culture show similar high expression by hCFs with low expression by hCECs. (**F_2_**) Expression of α-smooth muscle actin in hCF control. Imaged using a 40× objective lens. Scale bar = 10 μm (**C**,**D**), and 50 μm (**E**,**F**) [313].

**Figure 7:**
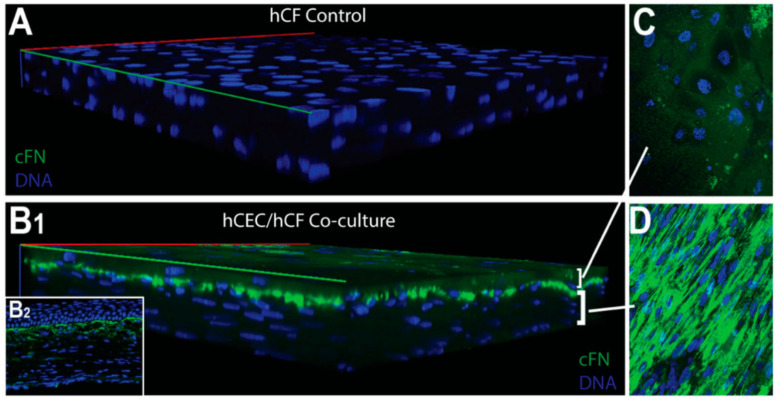
Fibronectin (cFN) expression in hCF stromal constructs and epithelial–stromal co-cultures. (**A**) hCF construct only, showing no fibronectin staining. (**B_1_**) Epithelial–stromal (hCEC/hCF) co-culture showing the expression of fibronectin at the epithelial–stromal interface, similar to (**B_2_**) rat cornea 1 week post-keratectomy. (**C**) Max projection image of the hCEC layer showing little expression in the epithelial layer. (**D**) Max projection image of the hCF layer showing high fibronectin expression at the epithelial–stromal interface. Imaged using a 40× objective lens [313].

**Figure 8:**
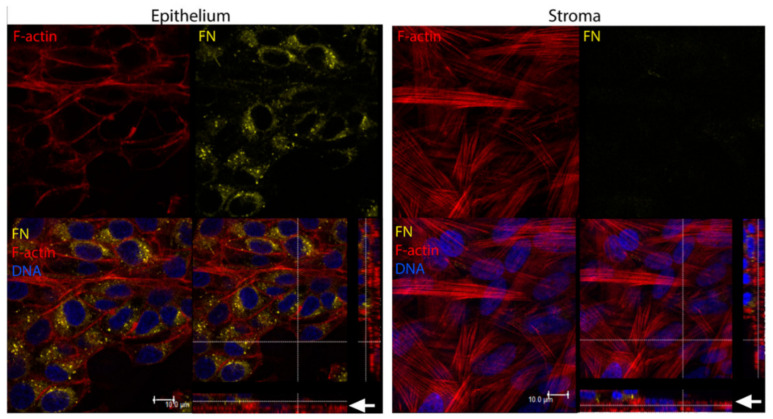
Localization of fibronectin (FN) in corneal epithelial–stromal co-cultures (hCE-TJ/hCF) at 3 days post-airlift. High expression of fibronectin in the epithelial layer (**left panel**) with little expression in the stromal layer (**right panel**). Arrows (white) denote the region of the epithelial–stromal interface. Scale bar = 10 μm [313].

**Figure 9:**
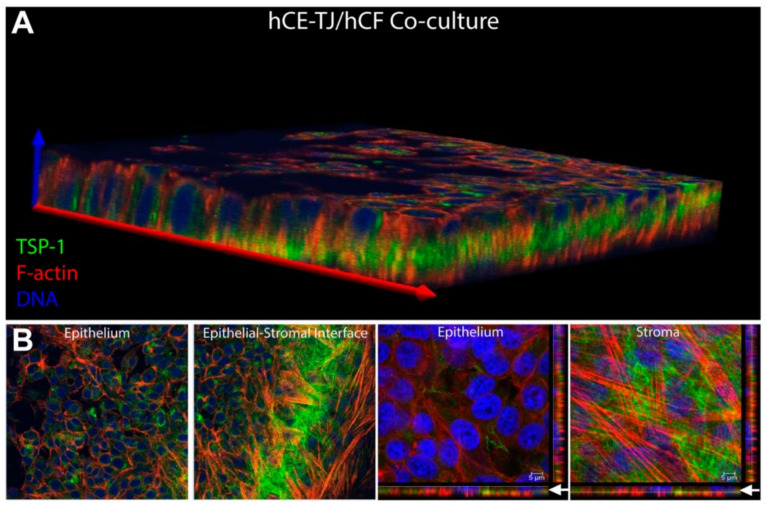
Thrombospondin-1 (TSP-1) expression in corneal epithelial–stromal co-cultures (hCE-TJ/hCF). (**A**) 3D-reconstruction of the corneal co-culture shows localization of thrombospondin-1 primarily at the epithelial–stromal interface. (**B**) Individual slices of the epithelial and stromal layers showed high expression within epithelial cells (left panel) and a fibrous appearance of thrombospondin-1 at the epithelial–stromal interface (right panel). Arrows (white) denote the region of the epithelial–stromal interface [313].

More recently, McKay et al. found that exosomes were released by human corneal epithelial cells, fibroblasts, and endothelial cells (Figure 4 and Figure 5), indicating the role of exosomes in the cell–cell signaling amongst these cell types [313]. The authors also reported the expression of α-SMA (Figure 6), fibronectin (Figure 7 and Figure 8), thrombospondin-1 (TSP-1) (Figure 9), and by human corneal epithelial cells, when co-cultured with corneal stromal fibroblasts [313]. This increased α-SMA expression and enhanced contractility suggest that epithelial exosomes promote stromal cell differentiation into myofibroblasts, which mediates wound closure [313]. The expression of the provisional matrix proteins, fibronectin and TSP-1, indicates that exosomes may also contribute to basement membrane regeneration and scar formation [313].

Wound healing properties of the exosomes from corneal stem cells have also been reported [308,309,314]. Samaeekia et al. investigated the effect of these exosomes on wound healing in vitro using a scratch assay, and in vivo using epithelial debridement wounds in mice [308]. They found that the exosomes that were derived from human corneal MSCs accelerated the corneal epithelial wound healing, both in vitro and in vivo, demonstrating their therapeutic potential in corneal epithelial wound repair [308]. Another study using a mouse model illustrated the therapeutic effects of MSC-derived exosomes on corneal wound healing [309]. Exosomes, via the transfer of miRNAs to corneal tissue, inhibited neutrophil invasion into the wounded area and promoted the regeneration of normal collagen in the corneal tissue [309]. These exosomes also attenuated the fibrotic scarring of the stroma, as measured by the reduced expression of the fibrotic genes encoding for collagen III and α-SMA [309]. When the packaging of miR159a into the exosomes was blocked by knocking out Alix protein, the exosomes with reduced miRNA were less effective at suppressing corneal scarring and promoting tissue regeneration [309]. These results demonstrate that corneal stromal stem cells exert wound healing effects through the miRNAs that are carried by exosomes [309]. Tao et al. induced an alkali injury in mouse corneas and found that exosomes that were derived from human placental MSCs increased the proliferation and migration of the corneal epithelial cells, and suppressed the inflammation, apoptosis, and angiogenesis at the wound site [314].

In a rabbit model, exosomes from human adipose-derived MSCs (ADSCs) significantly promoted the proliferation and suppressed apoptosis of corneal stromal cells [315]. In addition, the MMP expression was decreased, while extracellular matrix (ECM)-related proteins, such as fibronectin, were increased upon exosomal treatment [315]. These findings support the role of ADSC-derived exosomes in corneal stromal repair and ECM remodeling [315]. Similarly, in a mouse model of dry eye disease, a treatment with ADSC-derived exosomes ameliorated corneal epithelial damage, increased tear production, and decreased inflammatory cytokine proliferation [316]. Thus, ADSC-derived exosomes effectively inhibit NLRP3 inflammasome activation and reduce the corneal surface defects in dry eye disease [316]. In addition, the exosomes that are derived from MSCs and induced pluripotent stem cells (iPSCs) accelerate corneal wound repair [317]. However, iPSCs-derived exosomes exhibited significant effects on the proliferation, migration, and cell cycle promotion of human corneal epithelial cells, thus facilitating tissue regeneration and reducing corneal epithelial defects more effectively than the MSC-derived exosomes [317].

Recently, our group performed the first ever study that explored the role of SEs in corneal wound healing, by using primary corneal stromal cells from healthy (HCFs), type I diabetes mellitus (T1DMs), type II DM (T2DMs), and keratoconus (HKCs) subjects [318]. Scratch and cell migration assays were conducted at 0, 6, 12, 24, and 48 h after the SE stimulation (5 and 25 μg/mL) [318]. After the wound closure, the fibronectin was significantly downregulated in the HKCs, T1DMs, and T2DMs, with 25 μg/mL SE [318]. The HCFs, HKCs, and T2DMs showed a significant upregulation of thrombospondin 1, with 25 μg/mL SE [318]. The cleaved vimentin was significantly upregulated in the HKCs, T1DMs, and T2DMs, with 25 μg/mL SE [318]. These results highlight the potential therapeutic role of SEs in corneal wound healing and establish a strong foundation for future studies.

### 3.5. Autoimmune Uveitis

Autoimmune uveitis is an inflammation of the uvea, consisting of the iris, ciliary body, and choroid [319]. Its inflammatory response is mediated by pathogenic T cells and interleukins that breach the blood–retinal barrier and damage RPE cells [320,321]. RPE cells have been shown to release exosomes that aid in immunosuppression [322]. For instance, the exosomes from RPE cells that were stimulated with inflammatory cytokines (IL-1β, IFN-γ, and TNF-α) caused the apoptosis of monocytes, while the exosomes from RPE cells that were not stimulated with cytokines induced a pro-inflammatory CD14++CD16+ phenotype in human monocytes [322]. In addition, the stimulated RPE-derived exosomes significantly downregulated the T-cell proliferation, suggesting that RPE-derived exosomes may be able to suppress overactive inflammation in the retina by impeding the proliferation and infiltration of monocytes, as well as other inflammatory cells [322].

Similar to RPE-derived exosomes, MSC-derived exosomes also display immunoprotective effects in autoimmune uveitis [82,323,324]. Using a mixed lymphocyte reaction assay, Shigemoto-Kuroda et al. demonstrated that the exosomes that are secreted by bone-marrow-derived MSCs inhibit the inflammatory response of autoimmune uveitis, by suppressing the activation of antigen-presenting cells and Th1 and Th17 cells [323]. Umbilical cord MSC-derived exosomes were also found to alleviate autoimmune uveitis by blocking the chemoattractive effects of CCL2 and CCL21 on inflammatory cytokines [324]. Additionally, an in vitro study showed that umbilical cord MSC-derived exosomes had a slight inhibitory effect on interphotoreceptor retinoid-binding protein (IRBP)-specific Th17 responses, but significantly inhibited the Th17 responses that are mediated by dendritic cells [82].

Using a mouse model of experimental autoimmune uveitis (EAU), Kang et al. found that IL-35-producing regulatory B-cells (i35-Bregs) secrete exosomes that carry IL-35 [325]. After EAU was induced in mice, a treatment with the IL-35 exosomes resulted in the proliferation of IL-10- and IL-35-secreting T regulatory cells and the attenuation of Th17 responses, while marked choroiditis and photoreceptor cell death were seen in the control mouse eyes [325]. Even more recently, Jiang et al. examined the immunomodulatory role of circulating exosomes that were derived from uveitis rats that had been immunized with interphotoreceptor retinoid-binding protein R16 [326]. The treatment of non-immunized uveitis rats with these circulating exosomes blunted the inflammatory responses of the R16-specific T cells, as evidenced by a reduced IFN-γ and increased IL-10 production [326].

### 3.6. Uveal Melanoma

Uveal melanoma is a tumor arising from melanocytes [327], which are responsible for producing the pigment of the eye [328]. Uveal melanoma may be located at any point along the uveal tract, but more commonly involves the choroid than the iris and ciliary body [329,330]. It is the most common primary intraocular malignancy in adults, and those affected by the disease are not only at risk of losing their eyesight, but also their life [327,330]. Given the severity and prevalence of uveal melanoma, there have been several studies that have found exosomal biomarker candidates for the disease [257,331,332,333]. Ragusa et al. demonstrated the potential of miR-146a as a biomarker of uveal melanoma by detecting the increased expression of miR-146a in the serum-derived exosomes of uveal melanoma patients [257]. Eldh et al. found that exosomes that were isolated from the liver circulation of uveal melanoma patients contained Melan-A, indicating that these exosomes might be able to travel to the liver vasculature in metastatic uveal melanoma [333]. Interestingly, uveal-melanoma-derived exosomes exhibit a high expression of the proteins that are involved in focal adhesion, endocytosis, and the PI3K-Akt signaling pathway, such as heat shock protein (HSP) 90, HSP70, and integrin V [331], suggesting their involvement in cancer progression. Wroblewska et al. analyzed the proteomic profile of the serum-derived exosomes from primary and metastatic uveal melanoma patients [332] and found that the proteins that were involved in tumor development/metastasis, such as IFN-γ, interleukins 2, 11, and 12, and Pentraxin-3, were significantly elevated in the metastatic uveal melanoma exosomes [332].

### 3.7. Retinoblastoma

Retinoblastoma is the most common primary intraocular neoplasm in pediatrics, with most tumors occurring in infants and children younger than five years old [334]. The tumor formation occurs when both alleles of the retinoblastoma gene are mutated [334]. Retinoblastoma affects only one eye in approximately 75% of cases, involving spontaneous mutations of the retinoblastoma gene on chromosome 13q14 [334]. Bilateral disease is heritable in an autosomal dominant pattern, due to a germline mutation of the retinoblastoma gene [334]. The study of exosomes to determine the disease mechanisms of retinoblastoma is in its early stages. Castro-Magdonel et al. first examined the miRNA composition of the extracellular vesicles from children that were affected by retinoblastoma, and observed that miRNA-5787 and miRNA-6732-5p were significantly elevated in the extracellular vesicles and plasma of Rb patients [335]. Later, Ravishankar et al. compared the miRNA constituents of exosomes from the retinoblastoma cell lines, WERI-Rb-1 and NCC-Rb-51, versus the exosomes from a control cell line (MIO-M1). Their findings showed that miR-301b-3p and miR-216b-5p were elevated in the exosomes of both Rb cell lines [336]. The exosomes that were derived from the WERI-Rb1 retinoblastoma cells promoted tumor growth by invading the tumor microenvironment, increasing the proportion of tumor-associated macrophages, and decreasing the proportion of natural killer cells [337].

### 3.8. Proliferative Vitreoretinopathy

Proliferative vitreoretinopathy (PVR) is characterized by the proliferation of vitreous or retinal surface cells that form fibrotic membranes, and can result in tractional retinal detachment [338,339]. Similar to retinoblastoma, exosome-focused studies have only recently gained traction. Since the differentiation of RPE cells into mesenchymal cells via EMT has been associated with the early stages of PVR pathogenesis [338,340], Zhang et al. examined the role of exosomal miRNAs in EMT induction and PVR [341]. An in vitro model of PVR was created by using TGF-β2 to trigger the EMT of RPE cells, and these EMT-induced cells were co-cultured with normal recipient RPE cells [341]. The exosomes from the normal and EMT-induced RPE cells were extracted and analyzed, and 34 differentially expressed miRNAs were detected in the exosomes from the EMT-induced RPE cells [341]. Notably, miR-543 was found in the EMT-induced RPE exosomes and significantly induced the EMT of the recipient RPE cells, highlighting the role of exosomes in triggering PVR via EMT induction [341]. Interestingly, exosomal miR-4488 and miR-1273g-5p have been reported to inhibit the TGF-β2-stimulated EMT in ARPE-19 cells, by downregulating the ATP-binding cassette A4 (ABCA4) [258], which has been reported to be elevated in PVR tissue [342]. Overexpressed ABCA4 can counteract the inhibitory effect of miR-4488 and miR-1273g-5p on the proliferation, migration, and invasion of TGF-β2-stimulated ARPE-19 cells, suggesting that ABCA4-depleting therapies can slow PVR progression [258]. Overall, these studies demonstrate that exosomal miRNAs can both propagate and inhibit PVR fibrotic lesions [258,341].

### 3.9. Glaucoma

Glaucoma is the leading cause of global irreversible blindness, and the number of people with glaucoma worldwide is expected to increase from 79.6 million in 2020 to 111.8 million by 2040 [343,344]. Glaucoma is characterized by loss of retinal ganglion cells (RGCs), optic nerve atrophy, and the cupping of the optic disc, resulting in visual field defects and eventual blindness [345,346,347]. The primary risk factor for developing glaucoma is increased intraocular pressure (IOP), which is regulated by the outflow of aqueous humor, a fluid that is produced by the ciliary body epithelium and then drained into the systemic circulation via the trabecular meshwork [348,349,350]. The ciliary epithelium secretes exosomes containing RNA to the trabecular meshwork, and the trabecular meshwork releases exosomes back to the ciliary epithelium [351,352]. This exchange of translational signals helps to regulate IOP [351,352]. On the other hand, the disruption of this exosomal transfer may contribute to the development of glaucoma, when the production of aqueous humor is increased and the outflow of aqueous humor is decreased [353,354,355].

The presence of exosomes in aqueous humor has been reported and hypothesized to play a role in the IOP dysregulation in glaucoma [354]. Myocilin, a protein in aqueous humor that is bound to exosomes, may also contribute to the disease progression of glaucoma, since myocilin helps to clear the cell debris within the trabecular meshwork [353]. Mutations in myocilin are seen in glaucoma and lead to a blockage of the aqueous outflow via the trabecular meshwork, increasing the IOP [353]. In a study on the miRNAs that are associated with primary open-angle glaucoma, the levels of miR-182 expression were elevated in the trabecular-meshwork-derived exosomes and aqueous humor, suggesting that miR-182 may be a useful marker of primary open-angle glaucoma [356].

Several studies have revealed the immunoprotective roles of MSC-derived exosomes in glaucoma [357,358]. In a rat model, the addition of bone marrow MSC-derived exosomes to glaucomatous eyes attenuated the RGC atrophy and retinal nerve fiber layer thinning, demonstrating the neuroprotective effects of these exosomes [357]. Mead et al. also reported the neuroprotective effects of bone marrow MSC-derived exosomes in chronic ocular hypertension, showing that exosomes preserved the RGC function for up to 6 months [357]. In addition, exosomes from umbilical cord MSCs promoted the neuroprotection of RGCs in a rat optic nerve crush model [358].

The canonical Wnt signaling pathway is known to be involved in IOP regulation and possibly glaucoma [359,360,361,362]. Multiple studies have reported that non-pigmented ciliary epithelium (NPCE)-derived exosomes altered the canonical Wnt signaling in TM cells, decreasing their beta-catenin protein expression [352,363]. These findings suggest a therapeutic role of NPCE exosomes in glaucoma [352,363]. Specifically, it was shown that the miR-29b that was found in NPCE exosomes was responsible for the downregulation of the Wnt signaling, significantly reducing the levels of collagen III protein expression [351]. It is known that the trabecular meshwork of glaucoma patients is associated with the accumulation of collagen in the ECM and the upregulation of pro-fibrotic factors, such as TGF-β and α-SMA [364,365]. Therefore, upregulating the miR-29b that is carried by exosomes may serve as a useful therapeutic target to suppressing the collagen buildup in the ECM of the trabecular meshwork, normalizing the IOP levels in glaucoma patients [351].

**Table 3:** Exosomes in ocular diseases.

Disease	Exosome Type	Biomarkers	Effect	Reference
**Diabetic Retinopathy**	**Retinal cells**	Fibroblast growth factor, TNF-α, angiostatin miR-20a-3p, miR-20a-5p, miR-20b, VEGF	Pro-inflammatory, pro-angiogenic	[278,279]
		Circular RNA-cPWWP2A	Regulates endothelial cell activity via inhibition of miR-579	[289]
		miR-124-3p	Anti-inflammatory	[284]
	**RPE**	miR-202-5p	Anti-angiogenic	[286]
	**Plasma**	IgG	Damage to retinal endothelial cells by activating complement	[284]
				[280]
		miR-15a	Insulin production by pancreatic beta-cells, oxidative stress in T2D	[282]
	**MSCs**	miR-222	Retinal repair	[287]
**Age-Related Macular Degeneration**	**Serum**	miR-19a, miR-126, miR-410	Pro-angiogenic, retinal cell apoptosis	[308]
	**Retinal astroglial cells**	Endostatin, MMP-3	Anti-angiogenic, inhibit migration of macrophages and endothelial cells	[313]
	**RPE**	C3, CD63, CD81, LAMP2	Drusen production	[296]
		VEGF-2	Pro-angiogenic, retinal endothelial damage	[306]
		Cathepsin D, cytokeratins 8 and 14	Reduce oxidative stress	[305]
**Retinitis pigmentosa**	**Retinal cells**	PARP	Photoreceptor degeneration	[294]
**Retinoblastoma**	**Retinoblastoma cells**	miR-5787, miR-6732-5p, miR-301b-3p, miR-216b-5p, miR-92a-3p	Promote tumor growth and angiogenesis	[341,342]
**Corneal disease**	**Corneal stroma,** **Corneal epithelial cells**	Fibronectin, TSP-1, α-SMA	Cell migration, myofibroblast differentiation, wound closure	[316,319]
	**Corneal fibroblasts**	MMP14	Pro-angiogenic, load MMP2 into exosomes	[317]
	**Limbal stromal cells**	Keratin 15	Limbal epithelial cell proliferation via Akt phosphorylation	[318]
	**MSCs**	Col3a1, Acta2, Fibronectin	Corneal stromal repair	[315,321]
**Autoimmune Uveitis**	**RPE**	CD14, CD16	Anti-inflammatory, proliferation of IL-10 and T regulatory cells	[328]
	**Regulatory B-cells**	IL-35		[331]
	**Serum**	Retinoid-binding protein R16		[332]
**Uveal Melanoma**	**Liver vasculature**	Melan-A	Promote tumor growth and metastasis	[339]
	**Uveal melanoma cells**	HSP90, HSP70, integrin V		[337]
	**Serum**	Interferon-gamma, IL-2, IL-11, IL-12, Pentraxin-3		[338]
**Proliferative Vitreoretinoppathy**	**RPE**	miR-543	Induce the epithelial–mesenchymal transition (EMT) of recipient RPE cells	[347]
		miR-4488, miR-1273g-5p	Inhibit TGF-β2-stimulated EMT in RPE cells by downregulating ABCA4	[264]
**Glaucoma**	**Aqueous humor, Trabecular meshwork,** **Non-pigmented ciliary epithelium**	Myocilin, miR-182, miR-29b	Blockage of aqueous outflow via trabecular meshwork	[357,358,359,362]

Recently, Aires et al. found that stressing microglial cells with elevated hydrostatic pressure increased the microglial cell reactivity and retinal cell death, and upregulated exosome release by microglia [366]. Since microglial-cell-mediated inflammation occurs early in glaucoma progression, prior to RGC death, this indicates that the modulation of microglial function can alleviate the RGC degeneration [366].

## 4. Exosomes as Drug Therapies

**Figure 10:**
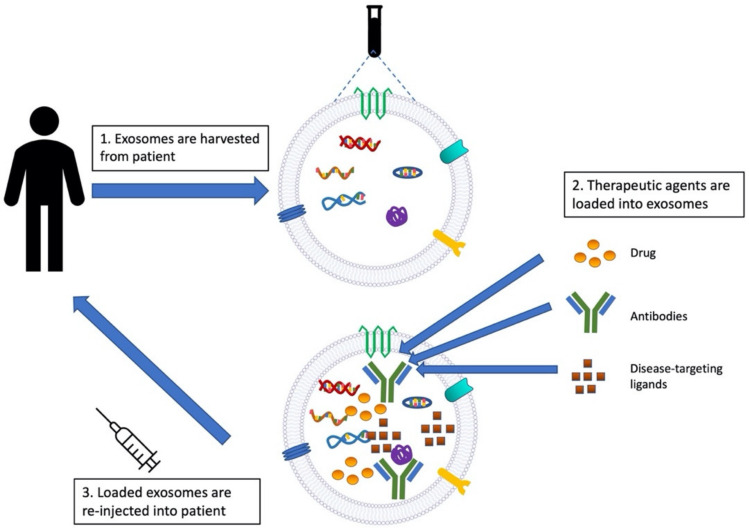
Using exosomes for drug delivery. Exosomes are first harvested from the patient. Then, therapeutic agents, including drugs, antibodies, and disease-targeting ligands, can be loaded into exosomes. Finally, the engineered exosomes are re-introduced into the patient.

The role of exosomes as drug carriers has gained interest due to exosomes’ versatility of function and composition, including bioactive molecules such as RNAs and proteins [11,12,13,14]. In particular, exosomes harbor tetraspanins such as CD63 on their membrane, to which drug molecules can bind with a high affinity, increasing the drug’s efficacy [11,15,367]. In addition, while synthetic drug vehicles suffer from the drawbacks of immunotoxicity and clearance by phagocytes, exosomes can cross the blood–brain barrier and other cell membranes [368,369]. The advantages of using exosomes as drug carriers include low rejection rates, the efficacy of the drug delivery, a prolonged drug activity, and a lower drug dosage required to have a therapeutic effect [3,370]. Exosomes can be utilized for drug delivery by first harvesting the exosomes from the patient, loading the exosomes with therapeutic agents or disease-targeting antibodies, and finally, reinjecting the loaded exosomes back into the patient [371], as displayed in Figure 10.

Exosomes are promising vectors for anti-tumor therapy due to their biocompatibility, low immunogenicity, and innate ability to interact with target cells [372,373,374]. Using exosomes as vehicles for paclitaxel, a drug that inhibits the microtubule assembly of cancer cells [375], has been shown to stabilize the drug concentrations in the plasma, allowing for a prolonged drug bioavailability and an increased drug efficacy [370,375,376]. Engineered exosomes have shown promising results as therapies for multiple cancer types [377,378]. For example, aptamer-modified exosomes targeting prostate cancer cells have effectively inhibited SIRT6 via siRNA delivery, downregulating the Notch and mTOR pathways that are involved in cancer signaling [377]. Exosomes carrying engineered HEK_293_T cells, which were transduced by a lentiviral vector chimeric gene, were shown to directly bind to the HER_2_/Neu receptors on breast cancer cells [378]. Upon binding to these HER_2_/Neu receptors, the exosomes transferred siRNA molecules to the breast cancer cells, downregulating the tumor protein D52 that was overexpressed by the breast cancer cells [378]. Recently, exosomes from normal fibroblasts that were transfected with Epstein–Barr-virus-induced 3 cDNA were electroporated with the siRNA of lymphocyte cytoplasmic protein 1 (LCP1) [379]. These engineered exosomes effectively transferred the siRNA of the LCP1 into OSCC cells, inhibiting their proliferation [379]. Sayyed et al. similarly demonstrated the ability of engineered exosomes to slow cancer progression [380]. Exosomes loaded with the miR-155 inhibitor improve the sensitivity of oral cancer cells to cisplatin, thus reversing miR-155-mediated chemoresistance [380].

Recently, MSC-derived exosomes have shown great promise as tumor-targeted therapies [381]. Using the novel gold nanoparticle labeling of exosomes and computed tomography imaging, Cohen et al. tracked exosomes that were derived from MSCs and the A431 squamous cell carcinoma line in A431 mice [381]. The MSC exosomes demonstrated the ability to selectively target the tumor and distribute throughout the tumor tissue and cytoplasm [381,382]. The MSC exosomes also showed a superior bioavailability, remaining in the bloodstream longer than the A431 exosomes [381]. Similarly, exosomes that were secreted from adipose-derived MSCs effectively inhibited tumor cell metastasis and apoptosis by transferring miR-145 to the breast cancer cells [382].

SEs as drug delivery vehicles have not yet been explored; however, the advantages of saliva as a source of exosomes make SEs a favorable vehicle for drug delivery [30,31,32]. SE collection is convenient and non-invasive, improving patient compliance [31,33]. Additionally, saliva does not coagulate, and SEs have been shown to be stable in biological fluids such as blood and gastric fluid [21,383]. SEs can therefore be easily obtained from the patient [31,33], injected with therapeutic agents, and re-introduced into the patient [384,385]. Using SEs for drug delivery thus has a great therapeutic potential in wide-ranging breakthrough applications, including for autoimmune diseases, neurodegenerative disorders, malignant neoplasms, and many others [386,387].

## 5. Conclusions

In summary, exosomes have a wide variety of effects on autoimmune diseases [52], neurodegenerative diseases [48,109], and malignant neoplasms [229]. Some notable trends are that MSC-derived exosomes have immunoprotective effects across these three disease categories, slowing the pathogenesis of inflammatory bowel disease [102], Alzheimer’s disease [149,150], oral squamous cell carcinoma [204], and breast cancer [222]. Additionally, SEs have been shown to accelerate the disease propagation in neurodegenerative diseases [22,48], whereas the effects of SEs in systemic autoimmune diseases [47,88] and cancer progression [209,243,255] have only recently been reported, and exhibit both proinflammatory and anti-inflammatory roles. However, the exosome biomarkers in these diseases can serve as useful biomarkers to aid in earlier detection of disease [47,88,209,243,255].

The impact of exosomes on the regulation of ocular diseases is profound and varied [302,313,323,332,366]. The biomarkers that are carried by exosomes possess great potential in monitoring the progression of ocular diseases [258,279,302,325,351]. The biomarkers that are transported by MSC-derived exosomes have exhibited mainly anti-inflammatory effects in diabetic retinopathy [279,280,281] and corneal disease [308,309,314], similar to the effects of MSC-derived exosomes in non-ocular diseases. In contrast, the biomarkers of other exosome types, such as serum-derived exosomes, have been revealed to both alleviate and exacerbate ocular disease pathogenesis [302,326,332].

Together, SEs hold great promise as a future target for numerous therapies, including ocular.

## Figures and Tables

**Table 1:** Advantages and disadvantages of exosomes derived from different sources.

Whole Saliva or Type of Non-SE	Disadvantage of Whole Saliva/Non-SE	Advantage of SEs	Shared Disadvantages of SEs and Non-SEs
Whole Saliva	Harbors contaminating elements and higher amylase enzyme levels	SE have a lipid bilayer that protects their cargo from contamination and degradation	
	Proteins from whole saliva are susceptible to degradation when removed from their natural environment		
Serum-derived exosome	Collection requires trained personnel and is often more invasive	SE collection is easy and non-invasive, improving patient compliance	Lack of methodologies to quantify contents of exosomes
	Coagulation poses challenges in handling these exosomes	Saliva does not coagulate	
Urinary exosome	Applications limited to kidney and prostate pathologies	Wide-ranging applications of SE, including systemic autoimmune disease, neurodegenerative disease, neoplasms, and ocular disease	
MSC-derived exosome	Rapid clearance from blood after administration in vivo	Stable in biological fluids	

## Data Availability

Not applicable.
